# Changes of bivalent chromatin coincide with increased expression of developmental genes in cancer

**DOI:** 10.1038/srep37393

**Published:** 2016-11-23

**Authors:** Stephan H. Bernhart, Helene Kretzmer, Lesca M. Holdt, Frank Jühling, Ole Ammerpohl, Anke K. Bergmann, Bernd H. Northoff, Gero Doose, Reiner Siebert, Peter F. Stadler, Steve Hoffmann

**Affiliations:** 1 Leipzig University, Chair of Bioinformatics, Leipzig, 04107, Germany; 2 Leipzig University, Transcriptome Bioinformatics Group - Interdisciplinary Center for Bioinformatics, Leipzig, 04107, Germany; 3 Ludwig-Maximilians-University, Institute of Laboratory Medicine, Munich, 81377, Germany; 4 Inserm, U1110 - Institut de Recherche sur les Maladies Virales et Hépatiques, Strasbourg, 67000, France; 5 Université de Strasbourg, Strasbourg, 67000, France; 6 Christian Albrechts University & University Hospital Schleswig-Holstein - Campus Kiel, Institute of Human Genetics, Kiel, 24105, Germany; 7 Christian Albrechts University Kiel & University Hospital Schleswig-Holstein - Campus Kiel, Department of Pediatrics, Kiel, 24105, Germany; 8 Ulm University & Ulm University Medical Center, Institute for Human Genetics, Ulm, 89081, Germany; 9 Leipzig University, LIFE - Leipzig Research Center for Civilization Diseases, Leipzig, 04107, Germany; 10 University of Vienna, Department of Theoretical Chemistry, Vienna, 1090, Austria; 11 Max-Planck-Institute for Mathematics in Sciences, Leipzig, 04103, Germany; 12 Santa Fe Institute, Santa Fe, NM 87501, USA

## Abstract

Bivalent (poised or paused) chromatin comprises activating and repressing histone modifications at the same location. This combination of epigenetic marks at promoter or enhancer regions keeps genes expressed at low levels but poised for rapid activation. Typically, DNA at bivalent promoters is only lowly methylated in normal cells, but frequently shows elevated methylation levels in cancer samples. Here, we developed a universal classifier built from chromatin data that can identify cancer samples solely from hypermethylation of bivalent chromatin. Tested on over 7,000 DNA methylation data sets from several cancer types, it reaches an AUC of 0.92. Although higher levels of DNA methylation are often associated with transcriptional silencing, counter-intuitive positive statistical dependencies between DNA methylation and expression levels have been recently reported for two cancer types. Here, we re-analyze combined expression and DNA methylation data sets, comprising over 5,000 samples, and demonstrate that the conjunction of hypermethylation of bivalent chromatin and up-regulation of the corresponding genes is a general phenomenon in cancer. This up-regulation affects many developmental genes and transcription factors, including dozens of homeobox genes and other genes implicated in cancer. Thus, we reason that the disturbance of bivalent chromatin may be intimately linked to tumorigenesis.

In recent years, a considerable amount of money and work has been spent to study cancer using modern high throughput sequencing experiments. However, especially when not only considering genomic, but also transcriptomic or epigenomic data, the evaluation and integration of the data is often missing. We here integrate thousands of DNA methylation and gene expression experiments from the International Cancer Genome Consortium^1^ (ICGC) and The Cancer Genome Atlas (TCGA)^2^ with chromatin state segmentations from the NIH Roadmap Epigenomics Project^3^ to assess the impact of bivalent chromatin in cancer.

Bivalent promoters and enhancers are abundant chromatin states in both, stem cells and differentiated cells^4^. They are characterized by the simultaneous enrichment of activating (e.g. Histone H3 lysine 4 monomethylation [H3K4me1] or trimethylation [H3K4me3]) and repressing (e.g. H3K27me3) chromatin modifications^5^. While the associated genes are repressed, bivalent promoters are pre-loaded with poised polymerase II (Pol II) to prepare genes for rapid activation^4^,^6^,^7^. Bivalent chromatin is frequently found within the promoter regions of developmentally important genes^8^,^9^. These regions have been suggested to “safeguard differentiation”^5^, and their malfunction might have a profound impact on the cell.

While DNA at bivalent promoters carries low levels of methylation in normal cells^3^,^10^, it was reported to be hypermethylated in cancer^11^,^12^,^13^,^14^,^15^. Accordingly, we showed in a recent study on the genome wide DNA methylation in two different sub-types of malignant lymphoma and normal controls, that the mean DNA methylation change at bivalent promoters of lymphoma samples is up to three times higher than in other chromatin state segments^16^. Unexpectedly, the majority of genes controlled by such hypermethylated bivalent promoters simultaneously showed increased expression levels in lymphoma samples. An up-regulation of genes controlled by bivalent promoters was recently shown in colorectal cancers^17^. However, it was reported that hypermethylation led to the continued repression of genes. Several other publications describe a continued repression of genes controlled by bivalent promoters via hypermethylation, suggesting that a loss of flexibility in gene expression contributes to tumorigenesis^15^,^18^,^19^. The vast amount of publicly available data allowed us to investigate whether the *positive* statistical dependence, or more informally *positive* correlation, of DNA methylation and gene expression is a common feature of different cancer types, or whether it is a lymphoma-specific phenomenon. Furthermore, we analyzed which types of genes are affected by this phenomenon.

## Results

### Distribution of bivalent chromatin in human cells

**Figure 1:**
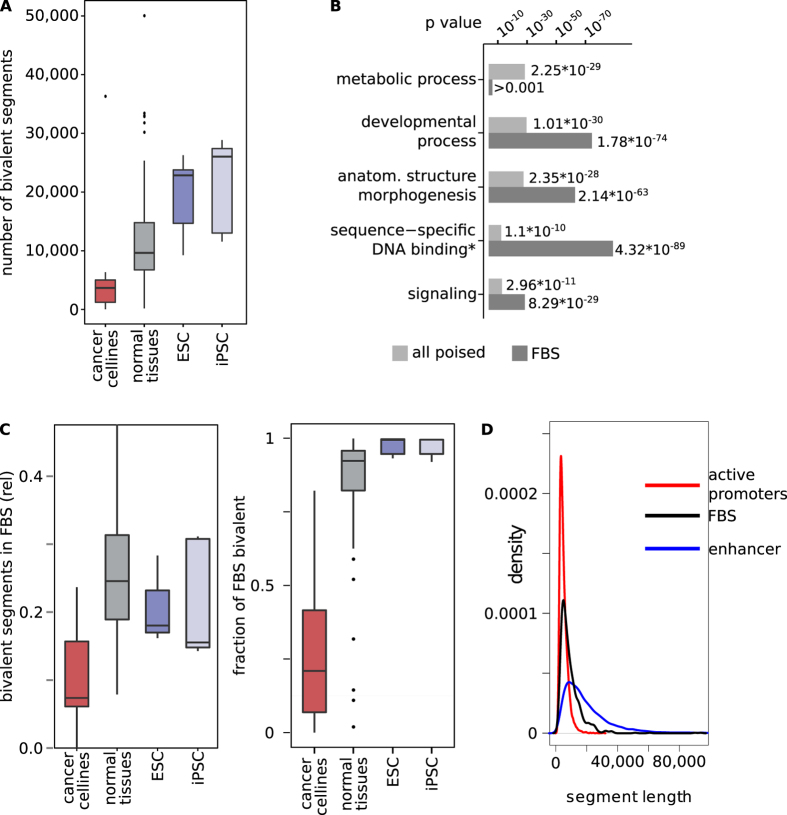
Bivalent chromatin segments in normal and cancer tissues. (**A**) Cancer cell lines show less bivalent regions than other types of cells. Number of bivalent chromatin segments in cancer cell lines (red), normal tissues or cells (grey), embryonic stem cells (ESC, blue) and induced pluripotent stem cells (iPSC, light blue). Stem cells on average have the highest number of bivalent segments, while cancer cell lines have low numbers of bivalent segments. (**B**) GO-term enrichment for genes associated to bivalent chromatin segments. Light grey: genes associated to any bivalent segment; Dark grey: Genes associated to a frequently bivalent segment (FBS genes). The enrichment of metabolic process genes is lost in FBSs, while the FBS genes are stronger enriched in the GO-terms developmental processes, anatomical structure morphogenesis, (*) sequence-specific DNA binding RNA polymerase II transcription factor activity and signaling. (**C**) FBSs well reflect normal bivalent chromatin states. Left: Fraction of bivalent chromatin segments that are part of a FBS in cancer cell lines (red), normal tissues or cells (grey), ESC (blue) and iPSC (light blue). On average, cancer cells are depleted in bivalent chromatin overlapping FBSs. Right: Fraction of FBSs that are bivalent in cancer cell lines (red), normal tissues or cells (grey), ESC (blue) and iPSC (light blue). FBSs are infrequently bivalent in cancer cell lines compared to normal cells. (**D**) Length distribution of FBSs (black) and segments based on active promoters (red) and enhancers (blue).

Chromatin state segmentations are maps identifying genomic intervals with distinct functional chromatin states, defined by characteristic combinations of histone modifications^20^. We analyzed 127 publicly available human chromatin state segmentations generated by NIH Roadmap (Supplementary Table S1) and 7 additional cancer cell line chromatin state segmentations (BLUEPRINT consortium^21^). We generated the chromatin state segmentations for the BLUEPRINT cell lines using the same method used by Roadmap. We confirmed that bivalent segments, i.e. bivalent promoters and bivalent enhancers, are present in all analyzed tissues. However, the total numbers differed strongly (Fig. 1A). As expected, embryonic stem cells (ESC) and induced pluripotent stem cells (iPSC) contained about twice as many bivalent segments on average compared to other tissues. Cancer cell lines, on the other hand, had the smallest number of bivalent segments. Differences of the same magnitude could not be observed for other chromatin state segments (see Supplementary Fig. S1A). In part, the usage of more heterogeneous tissue samples (as opposed to the homogeneous cancer cell lines) might explain this phenomenon. However, 60 of 109 of the differentiated tissue chromatin state segmentations were created using either cultured cells, cell lines or sorted blood cells that do not show the heterogeneity of tissue samples. Restricting the analysis to the 122 non-cancer chromatin state segmentations, and intersecting them with the Gencode v19 gene annotation^22^, we confirmed that genes associated with bivalent chromatin, i.e. genes with bivalent chromatin in their promoter regions or gene bodies, have a highly significant enrichment of the gene ontology (GO)-terms related to transcriptional activity and developmental processes, but also, e.g., to metabolic processes (see Fig. 1B, Supplementary Table S2).

### Frequently bivalent segments

To compare different cancer types, we were interested in genomic regions that have bivalent marks in the majority of cell types. We thus merged all non-cancer chromatin state segmentations of the NIH Roadmap, and identified 918 genomic regions that were bivalent in more than 80% (i.e. at least 98, frequently bivalent segments, FBSs) of normal cells. On average, 25% of the bivalent regions within a single data set overlapped FBSs (Fig. 1C). Recent publications used segments bivalent in ESC to study bivalent marks^11^,^13^,^23^,^24^,^25^. Since ESCs and iPSCs have a much higher number of bivalent segments than differentiated cells, FBS are designed to have a higher stability: a median of 87% of FBSs are overlapping bivalent segments in chromatin state segmentations of differentiated cells. In contrast, only 49% of a set of ESC derived bivalent segments overlap bivalent segments in differentiated cells (Supplementary Fig. S1B). However, all FBSs overlap a bivalent region in at least one ESC. The 80% requirement makes FBSs relatively robust against single erroneous segmentations. In the mean, FBSs span 9,300 nucleotides, which puts them between similarly defined active promoter (mean length = 5,000 nt) and enhancer (mean length = 20,000 nt) segments (Fig. 1D). Some FBSs spanning gene clusters such as the HOXA, HOXB and HOXC clusters, are up to 93,000 nt long. Among genes associated with FBSs (1,406 FBS genes), the enrichments of GO-terms related to transcriptional activity, developmental processes, and signaling were even stronger than among all bivalent segment genes. (cf. Fig. 1B, Supplementary Table S2).

### Chromatin bivalency is largely lost in cancer cell lines

In contrast to normal cells, bivalent segments in cancer cell lines showed a strong difference in number as well as their genomic locations. Cancer cell lines had fewer bivalent segments than all other chromatin types. Conversely, only an average of 28% of FBSs are bivalent in cancer cell lines, compared to 87% in normal cells (Fig. 1C), suggesting a disruption of chromatin bivalency in cancer cell lines. The strong enrichment of ESC-defined bivalent promoters that was shown for cancer associated promoters in fresh gastric cancer tissue^26^ supports this finding, since changing the state of bivalent promoters implies a loss of H3K4me3, and thus of bivalency.

**Figure 2:**
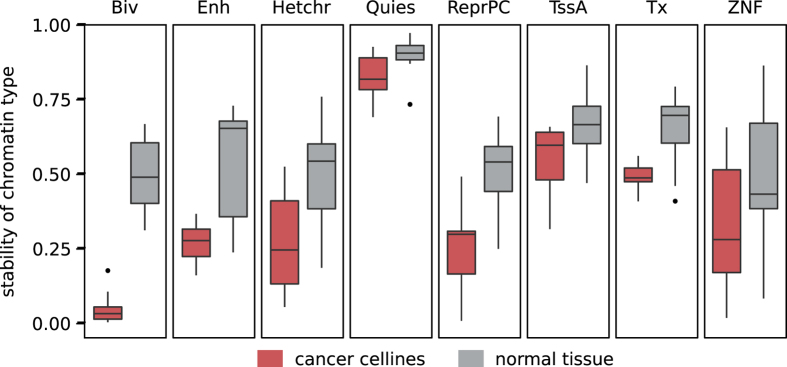
Cancer cell lines lose bivalent chromatin. Comparison of stability of chromatin state segments between related normal cells (grey) and cancer cell lines and their cells of origin (red). Stability described as relative number of nucleotides of the respective chromatin type where type is the same in both cells. Bivalent segments (Biv) are the least stable chromatin state segments in cancer cell lines, while they show average stability in related normal cells. Biv: Bivalent chromatin, Enh: Enhancer, Hetchr: Heterochromatin, Quies: Quiescent, TssA: Active promoter, Tx: Transcribed, ZNF: Zinc-finger/insulator, ReprPC: Polycomb repressed.

To analyze whether the loss of bivalent chromatin in cancer cell lines only reflects a general restructuring of the chromatin, we investigated the stability of chromatin types between related cell types. To this end, we paired the 11 segmentations of cancer cell lines with segmentations of their tissues of origin (subsequently called cancer cases). As a control, we created 11 pairs of closely related normal cells (subsequently called normal cases). As expected, the stability of most chromatin types was smaller in cancer cases than in the normal cases. However, the stability of bivalent chromatin in cancer cases was clearly the lowest when compared to all other states (Wilcoxon *p* < 10^−4^, Fig. 2). Likewise, bivalent chromatin showed a bigger difference in stability between cancer cases and normal cases than any other chromatin type (*z*-score 2.27). Recently, Hahn *et al*. described a “high instability or variability of bivalent promoters in colorectal cancer”^17^. Additionally, less detailed ChIP-Seq experiments on other fresh cancer tissue^21^,^26^,^27^ show that bivalent segments are less well conserved than active or repressed segments (Supplementary Fig. S2). In summary, these findings suggest that tumorigenesis is accompanied by a systematic disruption of bivalent chromatin marks in cancer.

### Hypermethylation of bivalent chromatin in bisulfite sequencing data

**Figure 3:**
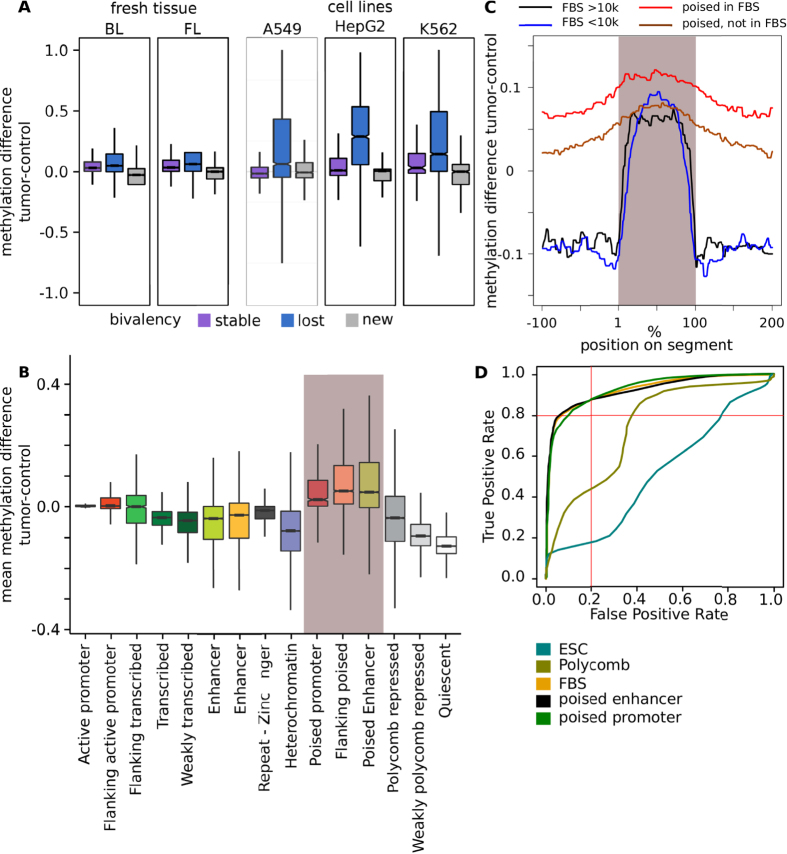
Hypermethylation of bivalent chromatin in cancer tissues. (**A**) Loss of bivalency is associated to hypermethylation. DNA methylation change of bivalent segments in relation to their stability. DNA methylation change for fresh cancer (left) and cancer cell lines (right) is shown for all elements in the cells of origin. Violet: chromatin bivalent in cancer and cell of origin, blue: chromatin no longer bivalent in cancer, grey: chromatin bivalent in cancer but not in cell of origin. (**B**) Bivalent chromatin regions show highest hypermethylation. DNA methylation difference for the 15 Roadmap chromatin states in BL. Bivalent states (grey box) show the strongest hypermethylation. (**C**) FBS regions are hypermethylated along their whole length. DNA methylation difference in BL at (grey marked area) and around FBSs, shown for FBSs > 10,000 nt (black), FBSs < 10,000 nt (blue), FBS sub-segments bivalent in primary B cells (red) and segments bivalent in primary B cells but not overlapping FBS (brown). (**D**) Methylation of bivalent regions is a good descriptor of cancer. ROC curve using relative descriptor methylation of FBSs (orange), bivalent enhancer (black), bivalent promoters (green), polycomb repressed regions without FBS (ocher) and ESC derived classifier (blue).

As DNA at bivalent chromatin is usually very lowly methylated, we suspected that the hypermethylation of bivalent chromatin in normal cells is accompanied by the loss of bivalency. A previous report on the correlation of hypermethylation with the loss of cancer related promoters in fresh gastric cancer^26^, is in line with this assumption. To further test this hypothesis, we compared the DNA methylation changes of chromatin state segments that lost their bivalent state in cancer cell lines to those that stayed bivalent or were only bivalent in cancer cell lines. This test included cell lines for five different types of cancer. In all data sets, hypermethylation of regions that lost their bivalency was significantly stronger (*p* < 10^−9^) than for stably bivalent regions, indicating that hypermethylation might indeed be associated with the loss of bivalency. On average, new bivalent regions did not show hypermethylation (Fig. 3A).

We re-analyzed the whole genome bisulfite sequencing (WGBS) data sets of Burkitt Lymphoma (BL) and Follicular Lymphoma (FL)^16^ to confirm genome wide hypermethylation of bivalent chromatin segments. Using a chromatin state segmentation of fresh primary B cells, we confirmed bivalent chromatin hypermethylation in lymphomas as compared to germinal center B cells. This was in contrast to the genome-wide loss of DNA methylation in BL (mean = −0.06) and FL (mean = −0.04) (Fig. 3B). Similar results were found for the WGBS data of acute lymphoblastic leukemia (ALL)^28^ and Medulloblastoma^29^ (Supplementary Fig. S3). Furthermore, in all but one case (Group 4 Medulloblastoma), bivalent states show the strongest hypermethylation of all chromatin states.

About a third of all FBSs are longer than 10,000 nucleotides, while bivalent regions in single chromatin state segmentations are much shorter, with less than 5% longer than 2,000 nt. We investigated whether the hypermethylation in cancer spans entire FBSs or is restricted to the shorter sub-segments that are bivalent in the cells of origin. Our analysis revealed that individual cell specific bivalent regions are frequently embedded in larger regions that are hypermethylated in cancer. In contrast, FBSs appear to better capture the entire regions that undergo hypermethylation. This is indicated by a sharp decrease of methylation level to the background level at the borders of FBSs (Fig. 3C, Supplementary Fig. S4), irrespective of the size of the FBSs. Thus, FBSs describe the borders of the regions of hypermethylation better than single bivalent regions do.

### Bivalent chromatin DNA methylation as a universal cancer marker

If the hypermethylation of bivalent segments and loss of bivalency was indeed a common phenomenon in cancer, it should be possible to distinguish cancer samples from normal tissue solely based on the DNA methylation levels of FBSs. To test this hypothesis, we considered the average relative DNA methylation level (see Methods) of the CpGs in the 14 FBSs that were bivalent in almost all (at least 120 of the 122) normal tissue segmentations in the Roadmap data set (Table 1). Note, that this approach works without a previous training step as is often used for DNA methylation classifiers.

Evaluating 450 k methylation array data from 6,347 cancers and 1,361 controls from various tissues (Supplementary Table S3), we found that the relative DNA methylation of these segments separated cancer and normal samples with high sensitivity and specificity (AUC = 0.926; Fig. 3D). Despite the high heterogeneity of cancer types and the relatively small number of 1,163 CpGs, their relative DNA methylation level provided a general and robust predictor of cancer (Table 2). Smaller subsets of the FBSs also identify cancer samples with consistently high AUC values (Supplementary Fig. S5, single FBS AUC in Table 1). In line with our results, previous attempts to classify individual cancers based on CpG methylation showed enrichment of bivalent genes in predictive areas^24^,^25^.

Our classifier overlapped with 24 protein coding genes, including 14 homeobox genes among which are seven HOX genes, two PAX genes (PAX3 and PAX6), LBX1, and DBX1. Additionally, it contained nine other genes involved in developmental processes, e.g. TBR1, ZIC2 and ZIC5. Many of these genes were frequently up-regulated in the ICGC cancer data (Supplementary Fig. S6).

To disentangle the impact exerted by bivalent enhancers and promoters, we repeated the classification with the most frequently bivalent promoters and enhancers, separately. These classifiers showed comparable performances, providing evidence that hypermethylation in cancer is a general phenomenon of bivalent chromatin, and is not restricted to promoters or enhancers (Fig. 3D). For all three classifiers, the lowest sensitivities and specificities were observed for renal carcinomas, stomach adenocarcinoma, and thyroid carcinoma, with an AUC ≥ 0.73. 16 types of cancer could be classified with high sensitivities and specificities (AUC > 0.9), of which 13, including lung and rectum carcinomas, even showed AUC > 0.95 (Table 2).

Additionally, we attempted to determine the tumor cell content expected to be necessary to predict cancer with this method. The estimated tumor cell content necessary for 95% true positive and 5% false negative rate varied from 27% in pancreas adenocarcinoma (PAAD) to about 77% in breast carcinoma (Supplementary Fig. S7, Supplementary Table S4). Correlations of descriptor methylation with tumor stage and grade were weak. We found a positive correlation to tumor stage (i.e. higher methylation in higher stages) in three cancer types (Kidney Renal Clear Cell Carcinoma (KIRC), Kidney Renal Papillary Cell Carcinoma and Head and Neck Thyroid Carcinoma), and negative correlation in Colon Adenocarcinoma. Histological tumor grades were available for seven cancer types. We found a positive correlation with grade in KIRC and PAAD (Supplementary Fig. S8, Supplementary Table S5). Nevertheless, correlations of the relative descriptor methylation with the tumor cell content were considerably higher (Supplementary Fig. S8A).

### Hypermethylation and up-regulation of genes as a global phenomenon in cancer

To test whether the association of bivalent segment hypermethylation with increased expression at the corresponding gene loci is a ubiquitous phenomenon in cancer, we evaluated expression using 5,545 RNA-Seq data sets and DNA methylation rates from 6,246 450 k arrays of 19 types of cancer and their respective controls published by ICGC and TCGA (Table 3). As some (9 of 19) cancers lack a chromatin state segmentation for their tissues of origin, we used FBSs for our analysis (we recall that on average, 87% of FBSs are bivalent in a chromatin state segmentation of a normal cell).

**Figure 4:**
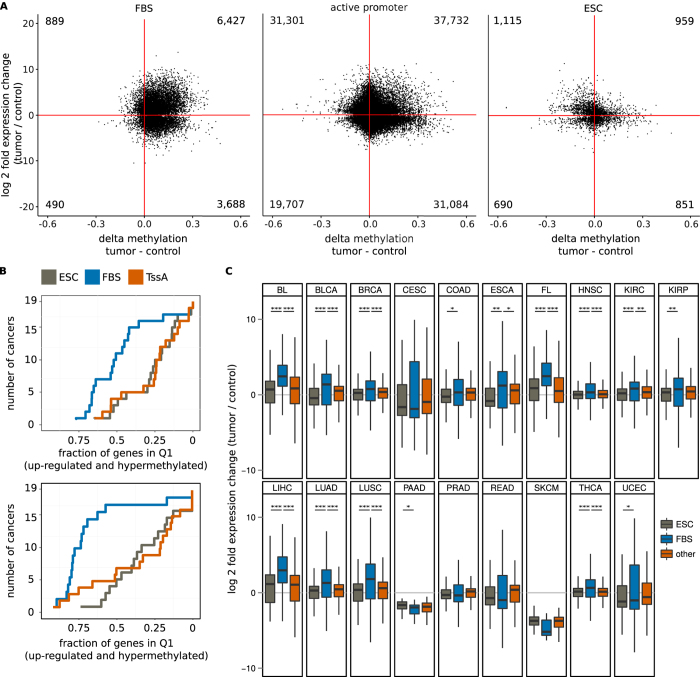
Up-regulation of bivalent chromatin genes in cancer tissues. (**A**) Scatter plots of DNA methylation change versus expression change for significantly differentially expressed FBS genes (left) and controls: active promoter background (center) and chromatin bivalent in ESC exclusively (right). (**B**) Cumulative plot of the number of cancers and gene/promoter pairs in quadrant one (Q1, hypermethylation and up-regulation) as the fraction of FBS genes (blue), ESC genes (grey) and background (red). Top: Only significantly differentially expressed genes are considered. Bottom: Only lowly and differentially expressed genes are considered. (**C**) Box-plot showing the expression change of FBS genes (blue), genes bivalent in ESC (grey) and other genes (red). Significance (Wilcoxon) of comparisons: ***p < 2.2 × 10^−9^, **p < 10^−3^, *p < 0.05. BL: Burkitt’s Lymphoma, BLCA: Bladder Urothelial Cancer, BRCA: Breast Cancer, CESC: Cervical Squamous Cell Carcinoma, COAD: Colon Adenocarcinoma, ESCA: Esophagus Cancer, FL: Follicular Lymphoma, HNSC: Head and Neck Squamous Cell Carcinoma, KIRC: Kidney Renal Clear Cell Carcinoma, KIRP: Kidney Renal Papillary Cell Carcinoma, LIHC: Liver Hepatocellular Carcinoma, LUAD: Lung Adenocarcinoma, LUSC: Lung Squamous Cell Carcinoma, PAAD: Pancreas Adenocarcinoma, PRAD: Prostate Adenocarcinoma, READ: Rectum Adenocarcinoma, SKCM: Skin Cutaneous Melanoma, THCA: Head and Neck Thyroid Carcinoma, UCEC: Uterine Corpus Endometrial Carcinoma.

We first investigated genes associated to FBSs (FBS genes) that were significantly differentially expressed. This analysis revealed a large degree of positive dependence between increased expression of FBS genes and increased DNA methylation: 15 of 19 cancers showed a significant up-regulation (mean log2 fold change: 0.94) and hypermethylation (mean change 0.07) in more than 40% of the FBS genes. To eliminate a possible bias due to the low baseline expression level of most FBS genes, we repeated the same analysis using only the subset of lowly expressed FBS genes to a background of lowly expressed genes. For these lowly expressed background genes, the same statistical problems as for lowly expressed FBS genes apply, eliminating possible biases caused by the low baseline expression. Again, in 17 of the 19 cancer data sets, lowly expressed FBS genes were mostly hypermethylated and significantly up-regulated (Fig. 4A,B, Supplementary Fig. S9). Compared to genes associated to genomic regions that are bivalent only in embryonic stem cells, FBS genes again showed a higher proportion of hypermethylated and significantly up-regulated genes in all but two cancer types.

Many RNA-Seq experiments showed a bias towards up-regulation of genes in cancer. To compensate for this effect, we compared the expression changes of FBS genes to all genes. In 11 of 19 cancers, the up-regulation of FBS genes was significantly stronger than that of the background, while none showed significantly weaker up-regulation (Fig. 4C). When restricting the analysis to lowly expressed genes, the number of cancer types showing significantly stronger up-regulation of FBS genes increased to 16. Comparisons to the genes bivalent in ESC did closely resemble these results.

#### Verification of FBS-gene up-regulation

**Figure 5:**
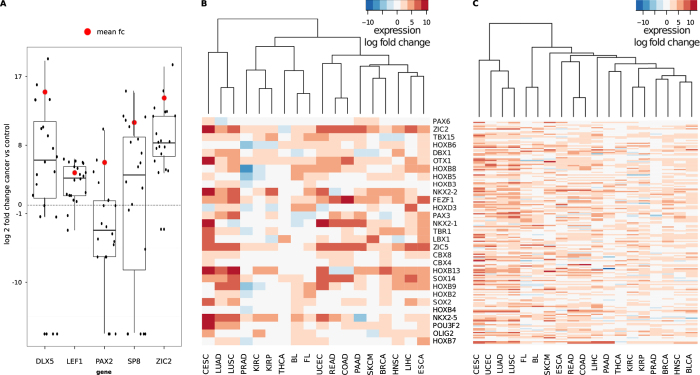
Expression of FBS genes. (**A**) Combined scatter and boxplots of expression differences in qPCR experiments for DLX5, LEF1, PAX2, SP8 and ZIC2 genes for 20 HCC cancer samples against normal liver tissue. The red dots show the log of the mean fold change, which is positive for all cancer experiments. (**B**) Heatmap of the expression log2 fold changes of selected FBS genes. Unsupervised clustering reveals expression similarities of FBS genes in related tissues. BL: Burkitt’s Lymphoma, BLCA: Bladder Urothelial Cancer, BRCA: Breast Cancer, CESC: Cervical Squamous Cell Carcinoma, COAD: Colon Adenocarcinoma, ESCA: Esophagus Cancer, FL: Follicular Lymphoma, HNSC: Head and Neck Squamous Cell Carcinoma, KIRC: Kidney Renal Clear Cell Carcinoma, KIRP: Kidney Renal Papillary Cell Carcinoma, LIHC: Liver Hepatocellular Carcinoma, LUAD: Lung Adenocarcinoma, LUSC: Lung Squamous Cell Carcinoma, PAAD: Pancreas Adenocarcinoma, PRAD: Prostate Adenocarcinoma, READ: Rectum Adenocarcinoma, SKCM: Skin Cutaneous Melanoma, THCA: Head and Neck Thyroid Carcinoma, UCEC: Uterine Corpus Endometrial Carcinoma. (**C**) Heatmap of expression changes for all FBS genes significantly up-regulated in more than 50% of the cancer data sets.

To verify the up-regulation of FBS genes, we chose five genes to perform qPCR on 20 samples of primary hepatocellular carcinoma (HCC) and corresponding normal liver tissue (Fig. 5A). Comparable to the RNA-Seq results for all FBS genes, this small subset showed considerable variability among samples. However, again reflecting the behavior of FBS gene expression in most cancers, we found a median increase of expression for four of the five genes. Focusing on the only exception, PAX2, we found that 12 samples showed down-regulation, two even a total loss of expression. In contrast, six showed significant up-regulation, three even a more than ten-fold increase of expression. Taken together, these results were in accordance with the overall picture we found for all FBS genes: most genes are up-regulated, albeit showing a high variability in expression values. Genes that showed overall down-regulation usually showed up-regulation in some samples. Accordingly, FBS gene up-regulation is not an artifact introduced by next generation sequencing, but a genuine biological effect that can be verified using alternative techniques.

### Analysis of up-regulated genes

To analyze which types of genes were mostly affected by up-regulation, we chose the 172 FBS genes that were significantly up-regulated in more than 50% of all cancers we studied. The requirement of significant differential expression in at least 10 cancer data sets was chosen to minimize the effect of possible expression outliers. We compared these up-regulated genes to the 68 FBS genes that showed a significant down-regulation in more than half of the cancer data sets (Supplementary Table S6). Even against a background of all FBS genes, the up-regulated genes are enriched with the GO terms for homeobox domains (*n* = 37, 1.6 fold enrichment) as well as in genes for transcription regulation (*n* = 55, 1.3 fold enrichment) and developmental proteins (*n* = 45, 1.3 fold enrichment). In contrast, down-regulated genes showed a depletion of homeobox containing genes (*n* = 2, 0.22 fold enrichment), developmental genes (*n* = 7, 0.5 fold enrichment) and transcription regulation genes (*n* = 15, 0.87 fold enrichment).

Unsupervised clustering of the expression fold changes of the FBS genes for all 19 cancer entities revealed similarities between cancers that originate from similar organs, such as lung squamous cell carcinoma (LUSC) and lung adenocarcinoma (LUAD) or rectum and colon adenocarcinoma (READ and COAD) (Fig. 5B,C). For the former pair, we observed a high expression of HOXC13, while the latter pair showed an up-regulation of SOX14. In addition, several members of the NKX homeobox family were frequently up-regulated. In particular, the cervix carcinoma produced high levels of NKX2-1, NKX2-2 and NKX2-5.

As homeobox genes have been shown to play an important role in both, oncogenesis and tumor suppression^30^, we further investigated the up-regulated genes of this gene family. In our analysis, HOXB13, previously associated with ovarian^31^ and breast carcinoma^32^,^33^, was strongly up-regulated in lung, skin, breast and gynecologic malignancies. OTX1, which has been connected to colon carcinogenesis and to non-hodgkin lymphoma^34^, was most strongly up-regulated in cervical carcinoma, but also very strongly in READ and COAD.

READ and COAD also transcribed increased amounts of the forebrain embryonic zinc finger protein 1 (FEZF1), which has been implicated in the development of gastric cancers^35^. Among the highly up-regulated genes, TBX15 showed the strongest hypermethylation across all cancer entities (median = 0.228). In the context of prostate carcinoma, the hypermethylation of TBX15 (as well as HOXD3) have been associated, via a positive correlation with ERG expression, to the histone deacetylase HDAC1, and a global epigenetic reprogramming^36^,^37^. Interestingly, two polycomb-group genes, i.e., CBX4 and CBX8, are among the genes that show significant up-regulation and hypermethylation in many cancer types. CBX8 has been connected to leukemogenesis^38^, while CBX4 potentiates angiogenesis in hepatocellular carcinoma^39^. Naturally, the extent of differential expression of the 172 genes comprising the set of frequently up-regulated FBS genes varies from cancer to cancer (Supplementary Table S7). However, 17 of the single cancer types, also had an enrichment of homeobox genes in the significantly up-regulated FBS genes. The two exceptions, PAAD and Skin Cutaneous Melanoma (SKCM), show very little significantly differentially expressed genes, due to the small number of control experiments.

### Evidence for lower expression of FBS genes in cell lines

**Figure 6:**
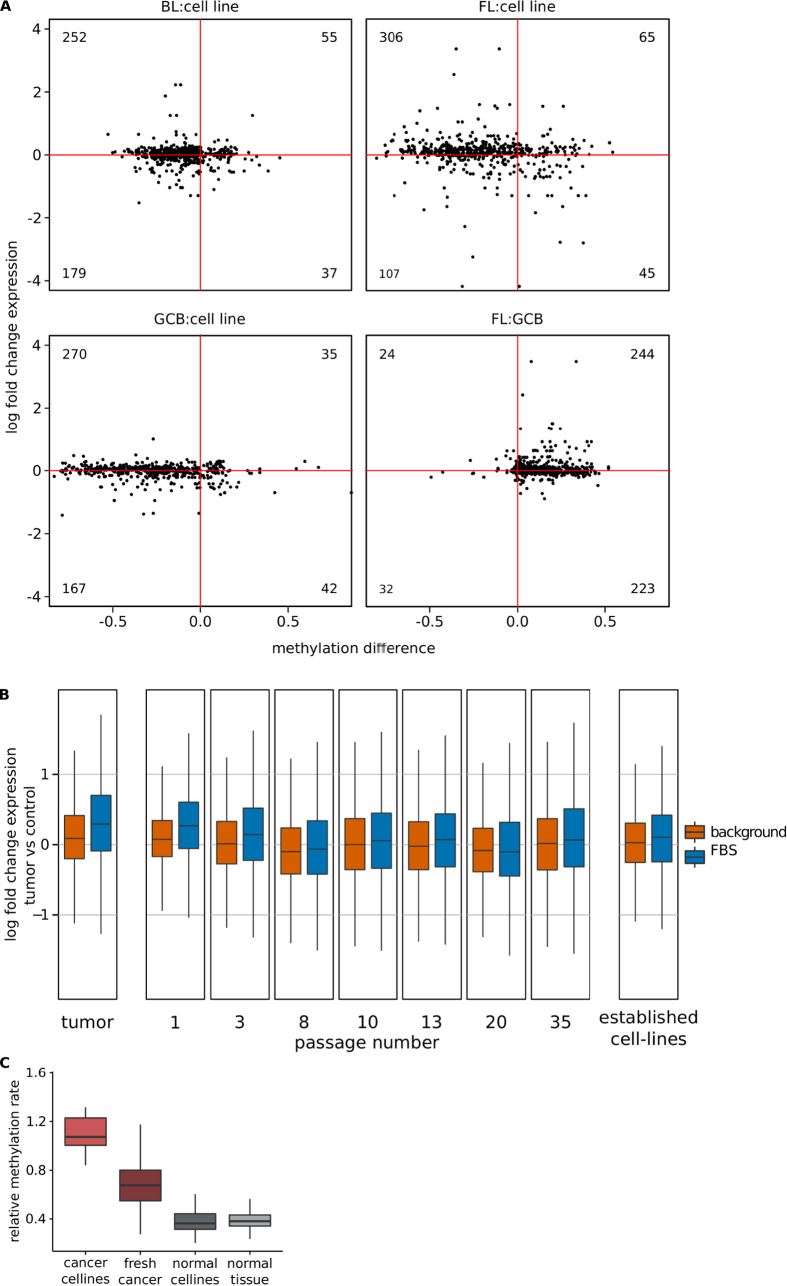
Expression and DNA methylation of cancer cell lines. (**A**) DNA methylation and expression changes of FBSs in BL cell lines against BL fresh tissues (top left), KARPAS-422 against FL fresh tissues (top right), lymphoma cell lines against normal germinal center B-cells (bottom left) and germinal center B-cells against FL fresh tissues. Changes are shown relative to the cell lines. The number of pairs in each quadrant is shown. (**B**) Expression change of FBS genes (blue) and other genes against normal cells in glioblastoma fresh tissue (left) and in cancer cell lines after a number of passages. At the right, the value for commonly used cell lines is shown. (**C**) Relative methylation of classifier FBSs in cancer cell lines, fresh cancer tissue, normal cell lines and normal tissue.

Earlier cell line based studies of hypermethylation in bivalent regions did not report an up-regulation of associated genes^15^. To investigate this discrepancy, we compared DNA methylation^16^ and gene expression^40^,^41^,^42^,^43^ of lymphoma cell lines to fresh cancer tissue and normal control cells. Indeed, our comparison revealed a hypermethylation of bivalent regions accompanied by a down-regulation of associated genes in the cell lines when compared to fresh cancer tissues (Fig. 6A). Our re-analysis of expression data of a glioblastoma cell line freshly generated by Lee *et al*.^44^ indicated that FBS genes are gradually silenced with each passage. After a couple of passages, FBS genes show an expression profile close to that in control cells and other established cell lines (Fig. 6B). We could confirm the repression of FBS genes in independent data from cervix carcinoma cell lines^45^ and also found a small but significant (*p* < 0.008) reduction of FBS gene expression in freshly generated cell lines from lung carcinoma metastasis^46^ (Supplementary Fig. S10). We also investigated whether the relative classifier CpG methylation of cell lines is different to the one of primary tissues. Cancer cell lines (but not normal cell lines) showed a significantly higher relative descriptor CpG methylation than fresh cancer tissues (*p* < 10^−13^, Fig. 6C).

## Discussion

In an attempt to reconcile our seemingly controversial findings we recall that (i) hypermethylation of bivalent elements is a frequent event in fresh cancer tissues. Our data indicates that (ii) this effect is even more pronounced in several cancer cell lines and that chromatin bivalency is frequently lost in cancer cell lines. While we see (iii) an up-regulation of associated genes in fresh cancers, (iv) their repression seems to be largely intact in the cell lines. We propose that the hypermethylation, the up-regulation, and the loss of bivalent chromatin might have a common cause, namely a genome wide malfunction of poising. Such a malfunction would lead to a deregulation of genes silenced by pausing, and a genome-wide “activation” of poised Pol IIs followed by a transient gene expression. Subsequently, promoter DNA methylation, loss of activating chromatin marks and other causes could gradually re-silence some genes, while other genes acquire sufficient gene body DNA methylation, recruit an alternative promoter or remain active due to other reasons. This would lead to a signal mixture of both, elevated expression and DNA methylation, in fresh cancer tissues. The fact that cancer cell lines show a significantly higher DNA methylation and an intact repression might suggest that these cell lines have largely managed to re-establish the silencing via DNA methylation. In fact, our results are compatible with a quick repression of FBS genes occurring during the creation of cell lines. Alternatively, cell lines might simply represent a less heterogeneous subset of the tumor cells, which would explain the lower number of up-regulated genes. However, this does not explain the overall gain in bivalent segment DNA methylation. Although our data indicates that bivalent marks are frequently lost in cancer, we could not determine a rule to predict the fate of bivalent elements in neoplastic diseases, and we have no data on the chain of events that leads to this loss. However, the process of transcription itself can lead to an exchange of histone H3/H4 tetramers^47^, which might facilitate the loss of the chromatin marks necessary for bivalency.

The mechanism that would lead to the activation of the Pol II also remains speculative. One possible trigger for the activation of poised Pol II is the proto-oncogene MYC. MYC is frequently over-expressed in cancer^48^, and facilitates Pol II pause release by recruiting the positive transcription elongation factor (P-TEFb), a pause release factor, to the paused Pol II^49^. Therefore, MYC has been predicted to cause expression of formerly bivalent genes in cancer^49^. However, the up-regulation bivalent genes is not limited to cancers over-expressing MYC (Table 4). The DNA hypermethylation observed may be explained by the enrichment of two DNA methyltransferases (DNMT1 and DNMT3b) surrounding bivalent promoters in cancer cell lines^50^. It is tempting to speculate that DNA methylation at formerly bivalent chromatin is a backup to re-enforce repression in case of a disruption of poising, emphasizing the importance of keeping the respective genes under tight control.

The tight control on the epigenetic level coincides with selective pressures on the genomic level. The epigenetic Roadmap project showed that bivalent chromatin tends to be enriched in non-exonic conserved elements (GERP)^51^ compared to its respective active chromatin counterpart^3^. This is another hint for the functional importance of bivalent chromatin. A change of chromatin state is essential for hypermethylation, since the presence of H3K4me3 would still protect the DNA from methylation.

Bivalent chromatin is known to play an important role in normal tissue development, but also seems to be crucial for keeping cells in their differentiated states. The enrichment of FBS associated homeobox genes (21.5% of 172) is indicative of this function. Homeobox genes such as the HOX family have been implicated to play an important role in both, oncogenesis and tumor suppression^30^. Abate-Shen described that homeobox genes exert their oncogenic effect via a strong over-expression or a temporospatially different expression pattern^52^.

Here, we show that the aberrant expression of developmental genes that are effectively no longer under the control of bivalent chromatin is present in various cancer types. In principle, such a runaway expression could lead to a de-differentiation of cells, much like the over-expression of the four Yamanaka factors used to generate induced pluripotent stem cells^53^. As an example, three out of four transcription factors (SOX2, POU3F2, and OLIG2) recently shown to be able to reprogram differentiated Glioblastoma cells to stem-like tumor-propagating cells^54^ are associated to a FBS. The three bivalent segments corresponding to these genes showed hypermethylation across all cancer types (median DNA methylation difference 0.13, 0.09 and 0.09, respectively), while their expression showed a median log2 fold change of 1.7, 1.8 and 1.2, respectively. The association of FBS genes to HDAC1 and SOX2 opens the question whether the disturbance of bivalent gene regulation could cause widespread epigenetic reprogramming. Such an epigenetic reprogramming has been suggested as a route how epigenetic alterations can contribute to tumorigenesis, by “compromising cellular differentiation pathways and facilitating the emergence of tumorigenic cells”^55^.

It has been shown that cancer risk factors, such as smoking or age, are correlated with the hypermethylation of CpGs at bivalent regions^24^. For dysplasia cells, however, also an age independent component was shown. Furthermore, the BL investigated here are pediatric cancers (median age 11 years), while the FL are mostly geriatric (median age 62 years). The fact that we see only little difference regarding bivalent chromatin DNA methylation and expression indicates that non age-related causes contribute to this effect.

Elucidating the molecular mechanisms behind this loss of the bivalent state has the potential to reveal novel preventive approaches against cancer. In the short term, measuring the DNA methylation of a small set of loci corresponding to frequently bivalent chromatin could provide a cost-effective and efficient universal cancer descriptor for diagnostic purposes. This test might be used globally to classify virtually all cancer types despite their cells of origin. The identification of frequently bivalent segments for special tissues can result in descriptors for sensitive and selective clinical diagnosis in types of cancer where other molecular descriptors are not yet available. Since bivalent marks are usually inferred from independent ChIP-Seq experiments, it cannot be ruled out that several bivalent marks annotated by the Roadmap Epigenomics project are artificial juxtapositions of H3K4me and H3K27me3 histone modifications. ChIP-reChIP experiments^8^,^56^ will help to improve the annotation of bivalent promoters and other chromatin state segments in the future.

## Methods

### Data Acquisition

We downloaded 127 15-state chromatin state segmentation annotations from the NIH Roadmap Epigenomics Project, including 16 chromatin state segmentations generated from ENCODE data.

(http://egg2.wustl.edu/roadmap/data/byFileType/chromhmmSegmentations/ChmmModels/coreMarks/jointModel/final/, all “15_coreMarks_mnemonics.bed.gz” files).

Normal and cancer RNAseq data were retrieved from TCGA. The RNAseq data comprised a total of 559 normal and 7,667 cancer data sets. BL, FL and GC-B data was published in ref. 16. The 450 k methylation data are taken from ICGC release 16^1^, TCGA, ENCODE^57^ and GEO^58^. The BL, FL, GC-B and BLUEPRINT data were published in ref. 16. This resulted in a total of 7,870 450 k cancer and 1,691 450 k normal cell data sets. Both, 450 k and RNAseq data for normal and cancerous tissue were available for 18 ICGC and one TCGA data set. A detailed list of accession numbers and IDs can be found in Supplementary Table S3.

#### Chromatin state segmentations

Bivalent segments were retrieved from 122 normal cell chromatin state segmentations from Roadmap. bedtools^59^ was used to merge their bivalent enhancer and bivalent promoter intervals, counting the number of distinct cell (or tissue) types that had contributed to each segment (using the count_distinct option of bedtools merge).

For the additional cancer cell line data, we used ChIP-Seq data (H3K27me3, H3K36me3, H3K4me1, H3K4me3 and H3K9me3) generated in the framework of the BLUEPRINT consortium^16^ that is publicly available for seven different blood cancer cell lines (BL2, DG-75, JVM2, Karpas-422, SU-DHL5, U-266, Z-138). To generate chromatin state segmentations for these seven BLUEPRINT cell lines that are comparable to the Roadmap segmentation, we downloaded Roadmap’s 15 state HMM model and used ChromHMM^60^ with this model to generate chromatin state segmentations out of the ChIP-Seq data files. Again, bedtools was used to gain merged bivalent chromatin state segmentations from bivalent enhancer and bivalent promoter segments.

### Frequently bivalent segments and associated genes

Frequently bivalent segments (FBSs) were required to be supported by a bivalent segment from more than 80%, i.e. at least 98, of the 122 Roadmap normal cell chromatin state segmentations. For comparisons, we defined a similar set of bivalent intervals soley based on ESCs to investigate whether FBS better capture bivalent segments in differentiated tissue or cells. To obtain this control set, we used all segments that were bivalent in at least 7 out of the 8 ESC chromatin state segmentations. A second control set to compare GO-term enrichments was obtained by merging intervals that are bivalent in at least three ESC, but in less than 12 normal cell chromatin state segmentations.

For the active promoter set, we first merged the active promoter segments from all 122 normal chromatin state segmentations using bedtools. Afterwards, all segments overlapping with FBSs were discarded.

For comparison of length distributions, we also generated data sets derived from merged active promoter and promoter flanking regions (“TssA”, “TssAflnk”) as well as from genic and normal enhancers (“Enh”, “EnhG”) in the same way used for bivalent chromatin segments. We defined genes that are associated to a chromatin segment as all genes where either the gene body or the promoter region overlapped the respective chromatin state segment. To get these genes, we used bedtools to intersect the respective segments with the Gencode v19 gene annotation. A region of 1,500 nt upstream was added to each gene annotation to capture the promoter regions.

For enrichment analysis of genes in GO-terms, KEGG pathways, and other descriptors we used GOrilla^61^, DAVID^62^ and string-DB^63^. In case of enrichment analysis with DAVID, we reported the Benjamini-Hochberg corrected p-values. Where not stated otherwise, all Gencode v19 genes were used as a background.

### Stability of bivalent segments in cancer

To assess the fate of bivalent segments in cancer, we first grouped related types of the 15 chromatin state segments together to create chromatin categories: “Bivalent” (Bivalent promoter, bivalent enhancer, flanking bivalent), “Promoter” (Active promoter, Flanking active promoter), “Transcribed” (Flanking transcribed, weakly transcribed, transcribed), “Enhancer” (Both types of Enhancer segmentation), “Polycomb repressed” (Polycomb repressed, weakly polycomb repressed), “ZNF”, “Quiescent” and “Heterochromatin”. We computed the stability of these chromatin categories in 11 cancer cell lines with respect to normal cells of origin, and compared them to nine pairs of related normal cells.

To assess the stability of each chromatin category, we used bedtools to intersect the chromatin state segmentations for every pair and determined the fraction of nucleotides that did not change categories relative to the normal segmentation. The pairs for cancer data sets and cells of origin were: Liver - HepG2; NHLF - A549; “Primary B from cord blood” - K562; “Primary T CD8+ naive cells from peripheral blood” - Dnd41 and “Primary B from peripheral blood” - BL2, DG75, JVM, KARPAS-422, SU-DHL, U266 and Z-138, respectively. For normal controls we used “Brain Cingulate Gyrus” - “Brain hippocampus Middle”; “Duodendum Mucosa” - “Colonic Mucosa”; “Fetal intestine large” - “Fetal intestine small”; “Fetal Muscle leg” - “Fetal Muscle Trunk”; HUES48 - HUES64; iPS20b - iPS18; “Primary T CD8+ naive” - “Primary T CD8+ memory”; “Primary hematopoietic stem cells G-CSF mobilized, Male” - “Primary hematopoietic stem cells G-CSF mobilized, Female”; “H9 Derived Neuronal Progenitor” - “H9 Derived Neuron cultured cells”; “hESC derived CD184+ Endoderm Cultured Cells” - “hESC derived CD56+ Ectoderm Cultured Cells”; “Foreskin Melanocyte Primary Cells skin 01”-“Foreskin Fibroblast Primary Cells skin01”.

For analysis of fresh cancer tissue, we downloaded ChIP-Seq peak files from BLUEPRINT (Blood), gastric tissue^26^ and breast cancer^27^ experiments.

For Blueprint data, we downloaded 5 ChIP-Seq broadpeak files (H3K4me3, H3K4me1, H3K9me3, H3K27me3 and H3K36me3) for four fresh cancers – two Acute Myeloid Leukaemia (AML) and two Multiple Myelome (MML) – as well as for naive B cells as controls. The peak files were segmented using the Roadmap 15 states model and lifted from hg38 to hg19 using the liftover tool of the UCSC genome browser. Stability was computed as described above.

Fresh tissue breast cancer ChIP-Seqs for H3K27me3 and H3K4me3 were downloaded from GEO (GEO:GSE40867). The peak files for the 9 patient samples were intersected with each other using bedtools to find bivalent domains. These bivalent domains and the peak files were intersected with the Roadmap segmentation for Breast-Myoepithelial cells, and stability was computed as before, with H3K4me3 used as “Promoter”, bivalent as “Bivalent” and H3K27me3 as “Polycomb repressed”. Gastric cancer data was downloaded from GEO (GEO:GSE51776) and fresh cancer was compared to the corresponding normal controls. Here, H3K4me1 was used as enhancer, H3K4me3 as promoter, H3K27 as repressed and the intersection of H3K4me1 or H3K4me3 with K27 methylation as bivalent enhancer and promoter, respectively. Stability was computed as described above.

### Methylation values from 450 K arrays and WGBS data sets

We first computed mean CpG-wise methylation rates for each tumor and its respective normal control by averaging over all 450 k-Array *β*-values of the respective group. Subsequently, we computed CpG-wise methylation differences by subtracting the mean methylation level of the control from the mean methylation level of the tumor. The resulting DNA methylation differences were intersected with the respective chromatin state segmentations using bigWigAverageOverBed of the UCSC tools^64^, and averaged within the segments. Segments that contained less than 3 covered CpGs (i.e. CpGs with methylation data) were discarded. Mean DNA methylation differences per chromatin state segment WGBS were calculated using bedtools merge. These mean methylation differences were analyzed using R.

We used the methylation differences described in Kretzmer *et al*.^16^ for BL, FL and GC-B data sets and intersected the DNA methylation differences with the chromatin state segmentation of “Primary B cells from peripheral blood”. The ALL data were taken from the GEO data set (GEO:GSE56601). We averaged the “ETV6-ALL difference from preB” and the “HD-ALL difference from preB” from this data set to calculate the DNA methylation differences. The averaged distances where then intersected with the “Primary B cells from cord blood” chromatin state segmentation using bigWigAverageOverBed, discarding segments with less than three covered CpGs. We used wiggletools^65^ to average the DNA methylation rates of the Medulloblastoma data set (ICGC:PBCA-DE) for the four distinct cancer groups Group3, Group4, SHH and WNT, and the control set. We also used wiggletools to compute the mean DNA methylation differences, which we intersected with the “Brain Substantia Nigra” chromatin state segmentation using bigWigAverageOverBed, again discarding segments with less than three covered CpGs.

For the analysis of the DNA methylation differences along the FBSs, we partitioned the FBSs segments by size, resulting in a group of FBSs below 10 k nt length and one above 10 k length. For comparison, we used bivalent regions of cell of origin chromatin state segmentations, split into those that did overlap an FBS and those that did not. Each single segment was extended by 100% of its length to the left and right and split into 300 sub-segments, each spanning 1% of the segment’s length. The mean DNA methylation difference for every sub-segment was computed using bigWigAverageOverBed. We averaged the mean DNA methylation for every sub-segment, discarding sub-segments without DNA methylation data. The data was plotted using R’s smooth function with standard parameters.

### DNA methylation changes of bivalent segments in cancer data

To investigate methylation changes in cancer, the Roadmap chromatin state segmentations of cancer cell lines, cancer fresh tissues and related normal tissues (A549:IMR90, K562:Primary B from cord blood, HepG2:Liver, BL:Primary B from periph blood, FL:Primary B from periph blood) were intersected. The segments were classified as “stable” when both, normal and cancer cell line, showed bivalent chromatin (bivalent enhancer, bivalent promoter or flanking bivalent), “lost” where only the chromatin state segmentation of the normal cell was bivalent, and “new” where only cancer showed bivalent chromatin. For DNA methylation analyses, WGBS (IMR90 and liver) and reduced representation bisulfite sequencing (RRBS) data (A549, K562, Primary B from cord blood and HepG2) from Roadmap were downloaded. For comparability, WGBS data sets were restricted to those CpGs that were present in the RRBS data sets. DNA methylation changes were computed using CpGs that had valid entries in both data sets, normal and cancer. The DNA methylation differences were analyzed using bigWigAverageOverBed and R.

### DNA methylation classifier

For the methylation based classifier we used those FBSs that had contributions from least 120 Roadmap single chromatin state segmentation data sets, i.e. 14 FBSs in total. Those FBSs cover 1,163 CpGs on 450 k arrays. Bivalent enhancer and bivalent promoter classifiers were constructed analogously by first merging only the bivalent enhancer or promoter segments, respectively, and then choosing the segments with most contributors. The classifier based only on bivalent promoters had nine segments supported by more than 97 chromatin state segmentations, corresponding to 322 CpGs on the 450 k array. The classifier based on bivalent enhancers consisted of 12 segments (more than 115 chromatin state segmentations) and corresponded to 706 CpGs on the 450 k array.

DNA methylation rates were computed using UCSC tools for ICGC data, converted to bigwig files and then analyzed using bigWigAverageOverBed and in-house perl tools for GEO and ENCODE array data. Classifier segments that covered less than three CpGs were discarded. We used the average of the group mean *β*-values of the segments to compute overall mean segment DNA methylation rates. Data sets with DNA methylation information for less than 3 segments were discarded. About 6,090 cancer and 1,300 control data sets met these criteria. For a classification of samples into cancer and healthy tissue, the relative CpG methylation in bivalent segments with respect to the mean methylation over all CpGs covered in the respective array, i.e. a methylation fold-change in bivalent segments, was used.

For comparison, the analysis was repeated using a data set comprising segments that are bivalent in all ESC and iPSC, but in less than three other more differentiated cells. This data set comprised 47 segments, but only six segments (=66 CpGs) were represented by more than two CpGs on the 450 k arrays. Therefore, we dropped this criteria and used all 92 CpGs present in any of the 47 segments for classification. In addition, the analysis was repeated on a data set for merged polycomb repressed (ReprPC) segments from which we subtracted all overlaps with a FBS using bedtools (212 CpGs).

“Receiver operator characteristics” (ROC) curves were plotted using R, “area under these curves” (AUCs) were computed using R’s AUC package^66^. Finally, we generated all 16,396 possible subsets of the 14 original classifier FBSs and compared their classification performance to the original classifier. A classifier based on only three FBSs performed best, reaching an AUC of slightly above 0.935.

#### Tumor cell content

We used infiniumpurify^67^ to predict tumor cell contents from the 450 k arrays. We only predicted tumor cell content for tumors where the descriptors were already provided by the infiniumpurify package. This analysis included the following cancer data sets: BLCA, BRCA, CESC, COAD, HNSC, KIRC, KIRP, LIHC, LUAD, LUSC, PAAD, SKCM, THCA and UCEC. We mapped tumor cell content information on relative descriptor methylation information and used R’s lm function to fit a linear model to the data points of every tumor separately. For every tumor where a linear model could be fitted, we determined at which purity value the lower bound of a 90% confidence interval prediction intersected the 5% false negative threshold. This value is given in Supplementary Table S4. Plots were constructed using R’s ggplot2 package.

#### Tumor stages and grades

We downloaded clinical metadata from TCGA and extracted pathologic stage and histologic grade information. To obtain sufficient power, several sub-stages needed to be merged. E.g. Stage Ia, Stage Ib and Stage Ic were summarized with Stage I. We mapped this information to the tumor cell content and relative descriptor methylation information. Correlations were computed between the relative descriptor methylation divided by tumor cell content and the tumor stages or grades using R’s cor.test function. For this, we mapped stages and grades to natural numbers: Stage I or G1 to 1, Stage II or G2 to 2, Stage III or G3 to 3 and Stage IV or G4 to 4. We discarded all other stages and grades. Plots were constructed using R’s ggplot2 package.

### Expression values

For RNA-Seq data from TCGA, we used raw gene read counts and edgeR^68^ with default parameters to compute fold changes and significance of expression differences (Supplementary Fig. S11). For array-based expression (GEO), we used logarithmic values, and all probes of a gene were averaged. The log expression values for each gene were averaged over the cancer and control groups, and the log2 fold change was computed.

Heatmaps of expression log fold changes generated from edgeR (Fig. 5) were generated using R’s heatmap.2 function with default parameters. Unsupervised clustering was performed by heatmap.2 using hclust in default settings.

### QPCR analysis

#### Ethics

Liver tissue samples were obtained from the Human Tissue and Cell Research (HTCR) Foundation^69^, which includes the informed patient's consent from all subjects. Experimental procedures were performed in accordance with the European Union-compliant ethical and legal framework of the Human Tissue and Cell Research (HTCR) Foundation. This framework has also been approved by the ethics commission of the Faculty of Medicine in the University of Munich and the Bavarian State Medical Association.

#### RNA isolation and qPCR transcript quantification

RNA from twenty paired samples of primary hepatocellular carcinoma (HCC) and corresponding noncancerous liver tissues (HTCR foundation, ref. 69) was isolated using the RNeasy Mini Kit (Qiagen) according to the manufacturer’s instructions. 2 *μ*g RNA was reverse transcribed according to published protocols^70^. Quantitative RT-PCR of distal-less homeobox 5 (DLX5), lymphoid enhancer binding factor 1 (LEF1), paired box 2 (PAX2), sp8 transcription factor (SP8) and zic family member 2 (ZIC2) was performed in quadruplicates using primers and probes from the human Universal ProbeLibrary (UPL. Roche) with AmpliTaq Gold (Thermo Fisher) (Supplementary Table S8). Gene expression changes were calculated using the ΔCt method as described previously^71^.

### DNA methylation vs. expression analysis of ICGC/TCGA data

To integrate changes in DNA methylation and changes in gene expression, the average DNA methylation differences for the FBS genes were plotted against the edgeR-derived log2 fold change expression of the associated genes. We used the active promoter set as described above and its associated genes as a background (Transcription start site active, TssA), as well as a data set that contained genes that overlapped with genomic regions that contained bivalent chromatin from at most 10 chromatin state segmentations, of which at least 3 were from embryonic stem cells. Subsets of genes significantly differentially expressed were computed by edgeR. Lowly expressed genes were defined as having a negative logCPM for the normal cells, computed by edgeR. In addition, the change in DNA methylation vs. change in expression was plotted for each cancer separately (Supplementary Fig. S7), again using bivalent ESC and active promoter genes as backgrounds.

### Cell lines

For lymphoma cell line analysis (Fig. 6A), we plotted expression vs. DNA methylation changes for BL, FL and GC-B data from ref. 16 in contrast to lymphoma cell lines (GEO access IDS in Supplementary Table S3). The mean values of BL-2 and DG75 cell lines were compared to BL, values of the diffuse large B-cell lymphoma cell line KARPAS-422 (cell line with typical BCL2 t(14;18) (q32;q21) translocation) was compared to FL values, and the mean values of BL-2, DG75 and KARPAS-422 were compared to GC-B. bigWigAverageOverBed was used to compute mean DNA methylation differences for the pairs stated above at merged bivalent chromatin state segments of “Primary B cells from peripheral blood”.

To compare expression changes of FBS and non-FBS genes (Fig. 6B) during the first passages of glioblastoma cell line generation, array based expression data (GEO:GSE4536) was re-analyzed. Average expression values for fresh tumor tissue, commonly used cell lines (T98, U87, U118, U138, U251, U373, U387), and newly generated cell lines at every available passage in both growing conditions (NBE, serum) seperately were calculated.

Furthermore, the expression changes of FBS genes were compared to the changes of non FBS associated genes for two types of lung tumor cell lines (GSE25251) as well as for the cervix data set (GSE9750). Box-plots of the log2 fold changes for the genes were generated using R’s ggplot. P-values were computed using R’s wilcox.test function.

### Statistical dependence and correlation

By statistical dependence we refer to the situation where the differential expression of a gene (first variable X) is *not statistically independent* from differential DNA methylation of the bivalent segment (second variable Y). Specifically, this association is *positive* if *P(X* > *x, Y* > *y*) ≥ *P(X* > *x*) *P(Y* > *y*) is satisfied. Thus, the *positive correlation* of two random variables is formally a special case of the positive dependence. However, in everyday language, statistical dependence is often referred to as correlation.

## Additional Information

**How to cite this article**: Bernhart, S. H. *et al*. Changes of bivalent chromatin coincide with increased expression of developmental genes in cancer. *Sci. Rep.* **6**, 37393; doi: 10.1038/srep37393 (2016).

**Publisher's note:** Springer Nature remains neutral with regard to jurisdictional claims in published maps and institutional affiliations.

## Supplementary Material

Supplementary Material

Supplementary Table 1

Supplementary Table 2

Supplementary Table 3

Supplementary Table 6

Supplementary Table 7

## Figures and Tables

**Table 1:** FBS classifier regions and genes associated.

Chr	Location	#data sets	ENSG	gene name	AUC
chr10	102979800–102995000	120	ENSG00000138136	LBX1	0.861
			ENSG00000227128	LBX1-AS1	
chr10	124898400–124914800	120	ENSG00000188816	HMX2	0.877
			ENSG00000154473	BUB3	
chr11	20175400–20187600	120	ENSG00000109851	DBX1	0.884
chr11	31817200–31852200	120	ENSG00000007372	PAX6	0.885
			ENSG00000049449	RCN1	
chr1	180197000–180213000	120	ENSG00000121454	LHX4	0.852
chr17	46616400–46706600	120	ENSG00000173917	HOXB2	0.697
			ENSG00000230148	HOXB-AS1	
			ENSG00000239552	HOXB-AS2	
			ENSG00000182742	HOXB4	
			ENSG00000257178	MIR10A	
			ENSG00000120075	HOXB5	
			ENSG00000120068	HOXB8	
			ENSG00000170689	HOXB9	
			ENSG00000108511	HOXB6	
			ENSG00000260027	HOXB7	
			ENSG00000242207	HOXB-AS4	
			ENSG00000120093	HOXB3	
			ENSG00000233101	HOXB-AS3	
chr17	48039800–48052200	120	ENSG00000199492	RNU6-1313P	0.821
			ENSG00000108813	DLX4	
chr2	162268600–162285200	120	ENSG00000136535	TBR1	0.914
			ENSG00000224076	AC009487.4	
			ENSG00000251621	AC009487.5	
			ENSG00000144290	SLC4A10	
chr2	175189800–175211200	120	ENSG00000217236	SP9	0.862
			ENSG00000268241	AC018470.1	
			ENSG00000231453	AC018470.4	
chr2	223152400–223173600	120	ENSG00000163081	CCDC140	0.899
			ENSG00000135903	PAX3	
chr4	113429200–113446000	120	ENSG00000178403	NEUROG2	0.888
			ENSG00000249509	RP11-402J6.1	
chr4	13523600–13551400	120	ENSG00000109705	NKX3-2	0.898
			ENSG00000246095	AC006445.8	
chr6	100050200–100063200	120	ENSG00000112238	PRDM13	0.907
chr13	100617800–100650800	121	ENSG00000139800	ZIC5	0.883
			ENSG00000043355	ZIC2	
			ENSG00000260738	LINC00554	

Location, number of contributing data sets, genes associated to the FBS classifier set and AUC when segment is used alone as a classifier.

**Table 2:** AUC of classifiers for the different ICGC and TCGA data sets.

Project	AUC bivalent promoter	AUC bivalent enhancer	AUC FBS
BL	0.928	0.883	0.9
BLCA-US	0.948	0.967	0.97
BRCA-US	0.925	0.958	0.963
CESC-US	1	1	1
COAD-US	0.982	0.984	0.979
ESCA	0.994	0.983	0.994
FL	0.945	0.923	0.921
HNSC-US	0.985	0.991	0.991
KIRC-US	0.987	0.847	0.944
KIRP-US	0.728	0.83	0.828
LIHC-US	0.92	0.904	0.92
LUAD-US	0.994	0.998	0.999
LUSC-US	0.995	0.995	0.998
PAAD-US	0.971	0.956	0.964
PRAD-US	0.893	0.911	0.909
READ-US	0.998	0.995	0.987
SKCM-US	0.965	0.968	0.978
STAD-US	0.824	0.73	0.794
THCA-US	0.875	0.867	0.885
UCEC-US	0.972	0.981	0.976
median	0.968	0.9625	0.967

**Table 3:** Number of ICGC and TCGA data sets used.

Project	Meth. t	Meth. n	Exp. t	Exp. n	Cancer name
BL	32	16	18	5	Burkitt’s Lymphoma
BLCA-US	198	19	405	19	Bladder Urothelial Cancer
BRCA-US	662	96	766	88	Breast Cancer
CESC-US	124	3	296	3	Cervical Squamous Cell Carcinoma
CLLE-ES	58	0	—	—	Chronic Lymphocytic Leukemia
COAD-US	271	37	282	41	Colon Adenocarcinoma
ESCA	174	13	173	10	Esophagus Cancer
FL	26	16	46	5	Follicular Lymphoma
GBM-US	59	0	—	—	Brain Glioblastoma Multiforme
HNSC-US	376	50	261	31	Head and Neck Squamous Cell Carcinoma
KIRC-US	285	158	472	68	Kidney Renal Clear Cell Carcinoma
KIRP-US	132	44	289	32	Kidney Renal Papillary Cell Carcinoma
LAML-US	170	0	—	—	Acute Myeloid Leukemia
LGG-US	297	0	—	—	Brain Lower Grade Glioma
LIHC-US	140	44	16	9	Liver Hepatocellular Carcinoma
LUAD-US	395	28	124	37	Lung Adenocarcinoma
LUSC-US	277	42	224	17	Lung Squamous Cell Carcinoma
OV-AU	116	0	—	—	Ovarian Cancer
PAAD-US	69	2	483	4	Pancreas Adenocarcinoma
PAEN-AU	34	0	—	—	Pancreatic Cancer Endocrine neoplasms
PRAD-US	198	46	483	51	Prostate Adenocarcinoma
READ-US	94	7	91	10	Rectum Adenocarcinoma
SKCM-US	332	2	104	1	Skin Cutaneous Melanoma
STAD-US	245	2	—	—	Gastric Adenocarcinoma
THCA-US	494	53	500	57	Head and Neck Thyroid Carcinoma
UCEC-US	364	34	329	5	Uterine Corpus Endometrial Carcinoma
total	5622	696	5362	488	

Meth. t: number of tumor 450 k methylation arrays used. Meth.n: number of control 450 k methylation arrays used. Exp. t: Number of tumor RNA-Seq data sets used. Exp. n: number of control RNA-Seq data sets used.

**Table 4:** Log2 fold changes for MYC and N-MYC.

cancer	N-MYC	MYC
BLCA	2.08	−1.62
BL	1.70	3.60
BRCA	1.75	−0.98
CESC	2.30	−0.14
COAD	2.80	1.97
ESCA	0.99	0.55
FL	4.59	0.36
HNSC	0.21	−0.42
KIRC	−1.91	1.58
KIRP	−2.74	1.15
LIHC	3.32	−0.81
LUAD	3.41	−0.13
LUSC	3.77	0.30
PAAD	0.74	−0.35
PRAD	−0.61	0.83
READ	1.90	1.94
SKCM	1.54	0.97
THCA	−0.93	−0.75
UCEC	4.44	−1.26
