# Supplementary material for: Changes of bivalent chromatin coincide with increased expression of developmental genes in cancer
Source: Sci Rep. 2016 Nov 23;6:37393. doi: 10.1038/srep37393 (PMC5120258; doi:10.1038/srep37393)
Supplement: Supplementary Material [file srep37393-s1.pdf]

Changes of bivalent chromatin coincide with increased expression of developmental genes in cancer

Stephan H Bernhart, Helene Kretzmer, Lesca M Holdt, Frank Jühling, Ole Ammerpohl, Anke K Bergmann, Bernd Northoff, Gero Doose, Reiner Siebert, Peter F Stadler, Steve Hoffmann

Supplementary Figures

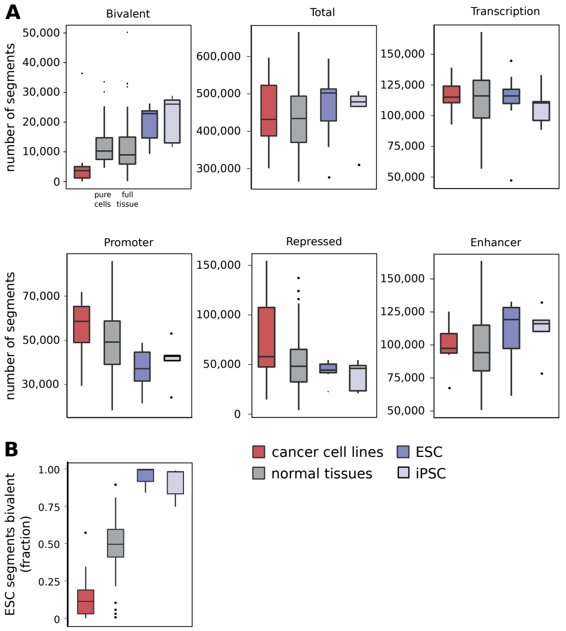

Supplemental Material, Figure S1: **A** Number of chromatin intervals of bivalent (TssBiv, BivFlnk and EnhBiv, top left), total number of chromatin intervals (top center), Transcription intervals (TxFlnk, Tx and TxWk, top right), Promoter intervals (TssA and TssAFlnk, bottom left), Repressed intervals (ReprPC and ReprPCWk, bottom center) and Enhancer intervals (Enh and EnhG, bottom right) divided in cancer cell lines (red), normal tissue (grey), ESC (blue) and iPSC (light blue). In bivalent intervals normal tissue is subdivided into contributions from pure cells (left) and from whole tissues (right). **B** Fraction of ESC derived bivalent regions that are bivalent in cancer cell lines (red), normal tissue (grey), ESC (blue) and iPSC (light blue).

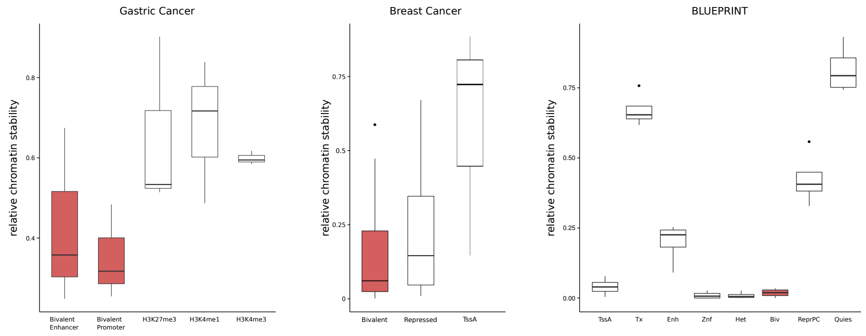

Supplemental Material, Figure S2: Stability of bivalent chromatin (red) in fresh cancer tissue. Right: Gastric cancer, stability of activating and repressing marks and bivalent marks (red). Center: Stability of TssA (TssA and TssAFlnk) and Repressed (ReprPC) compared to Bivalent (TssBiv) segments in breast cancer tissue. Right: Stability of segments in BLUEPRINT fresh cancer tissue, compared to naive B-cells. Note that the chromatin marks are not statistically independent.

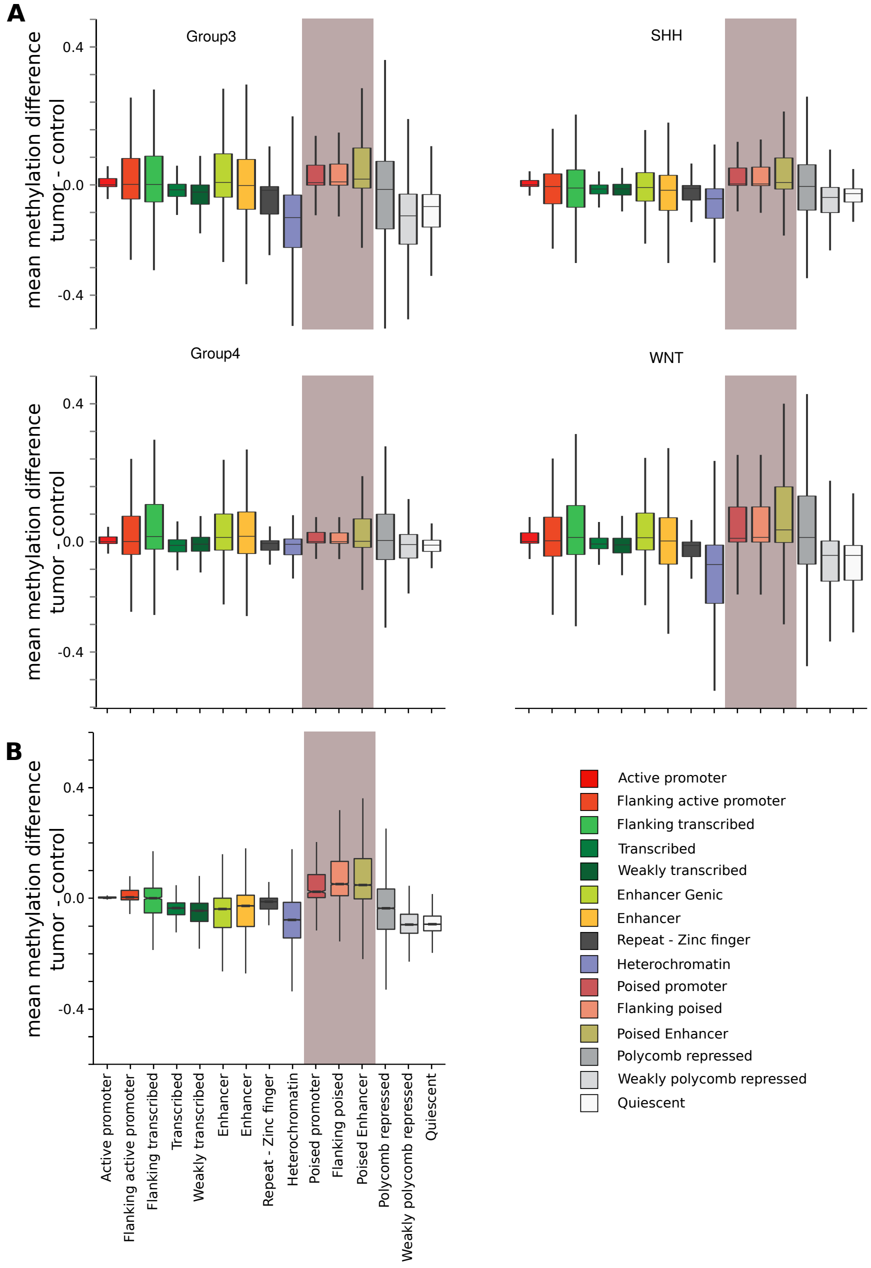

Supplemental Material, Figure S3: Methylation changes in **A** Medulloblastoma sub-types: Group 3 top left, SHH (Sonic hedgehog) top right, Group 4 bottom left and WNT bottom right. **B** ALL in the chromatin segments of the respective cells of origin ("Brain substantia nigra" and "Primary B cells from cord blood").

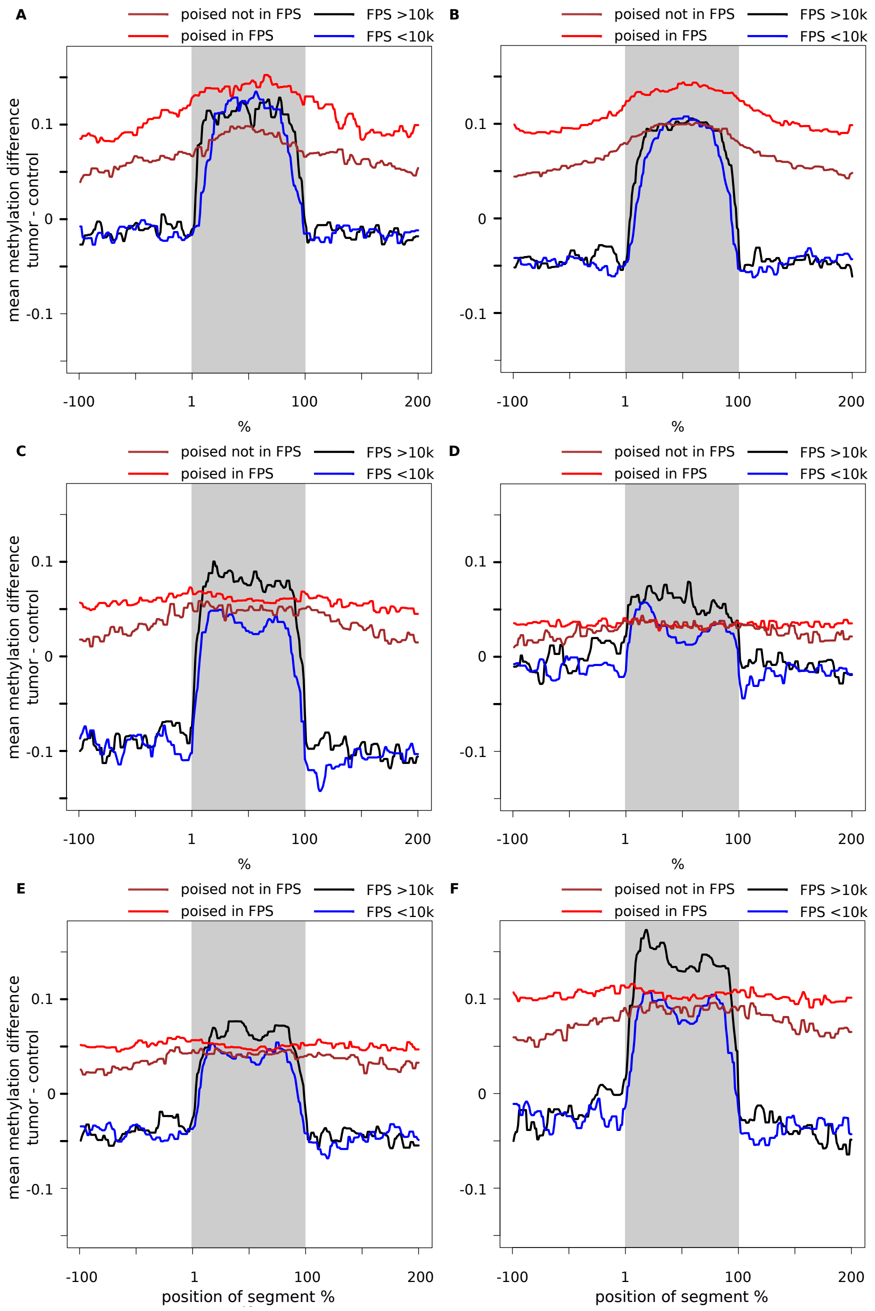

Supplemental Material, Figure S4: Averaged methylation changes for long FBS (>10k, black), short FBS (<10k, blue) and bivalent segments within FBS (red) and not within FBS (brown) for **A** ALL, **B** FL, **C** Group 3 medulloblastoma, **D** Group 4 medulloblastoma, **E** SHH-type (Sonic Hedgehog) medulloblastoma and **F** WNT-type medulloblastoma.

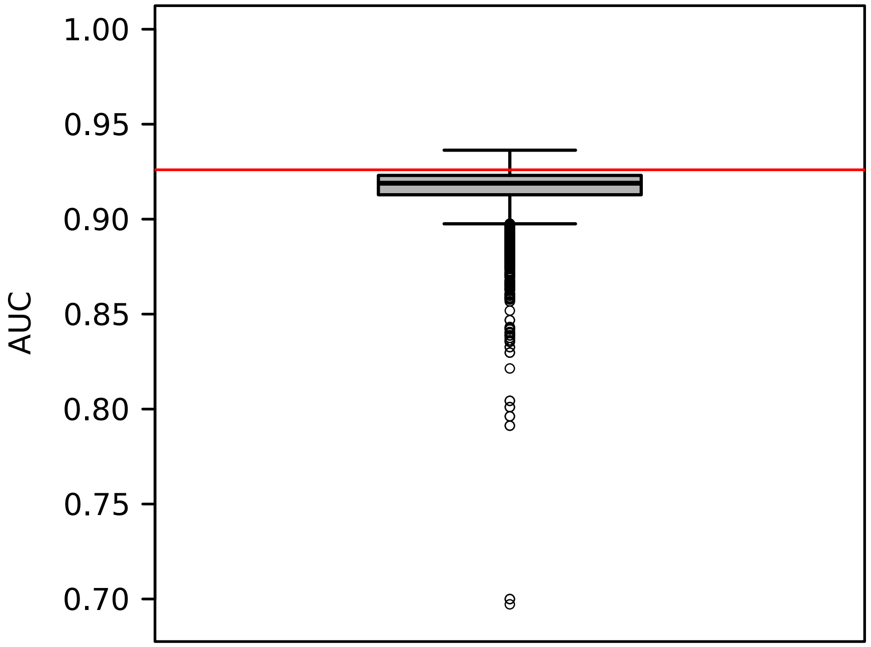

Supplemental Material, Figure S5: AUC values for all subsets of segments of the FBS classifier. Red line at 0.926, the AUC of the full classifier. Maximum AUC (0.936) was achieved by a combination of descriptor FBS 4 (PAX6, RCN1), 9 (SP9), and 14 (ZIC5,ZIC2)

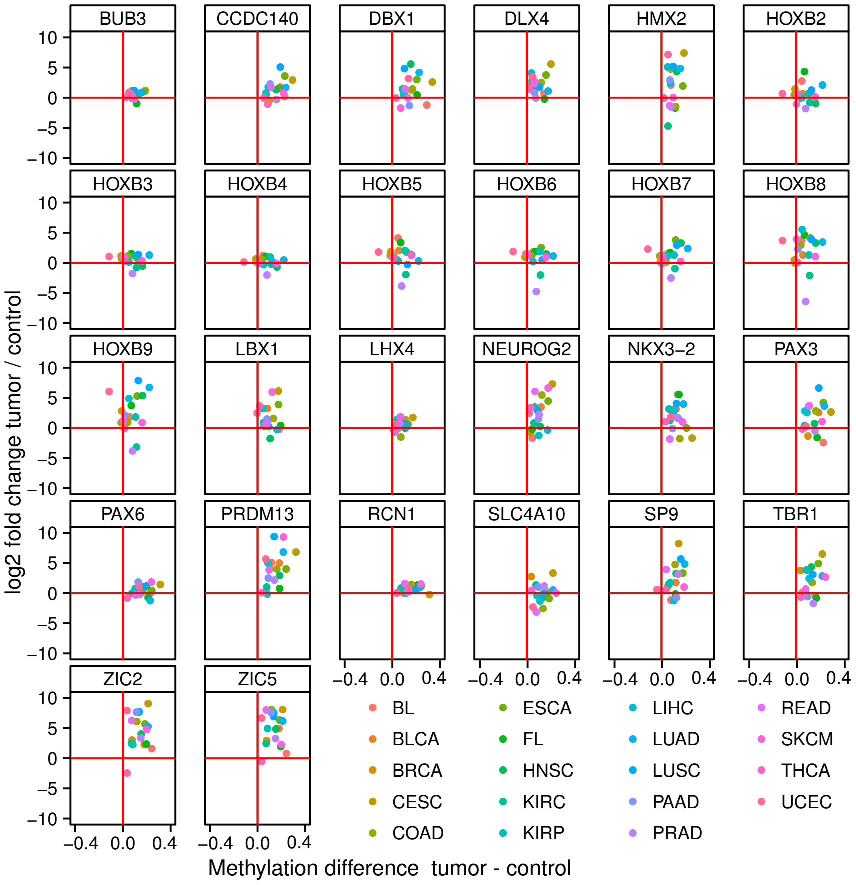

Supplemental Material, Figure S6: Correlation of expression and bivalent segment methylation in cancer for bivalent segment classifier genes.

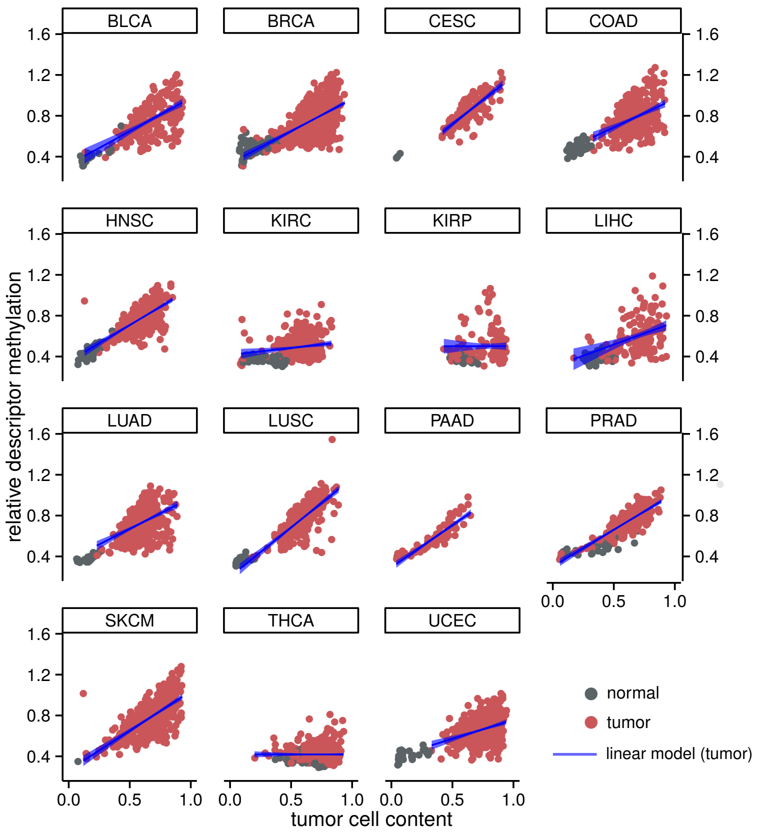

Supplemental Material, Figure S7: Correlation of relative descriptor methylation and tumor cell content for 15 different cancer sets. Grey points: normal, red points: tumor. A linear model (blue) fitted to the tumor points is also shown.

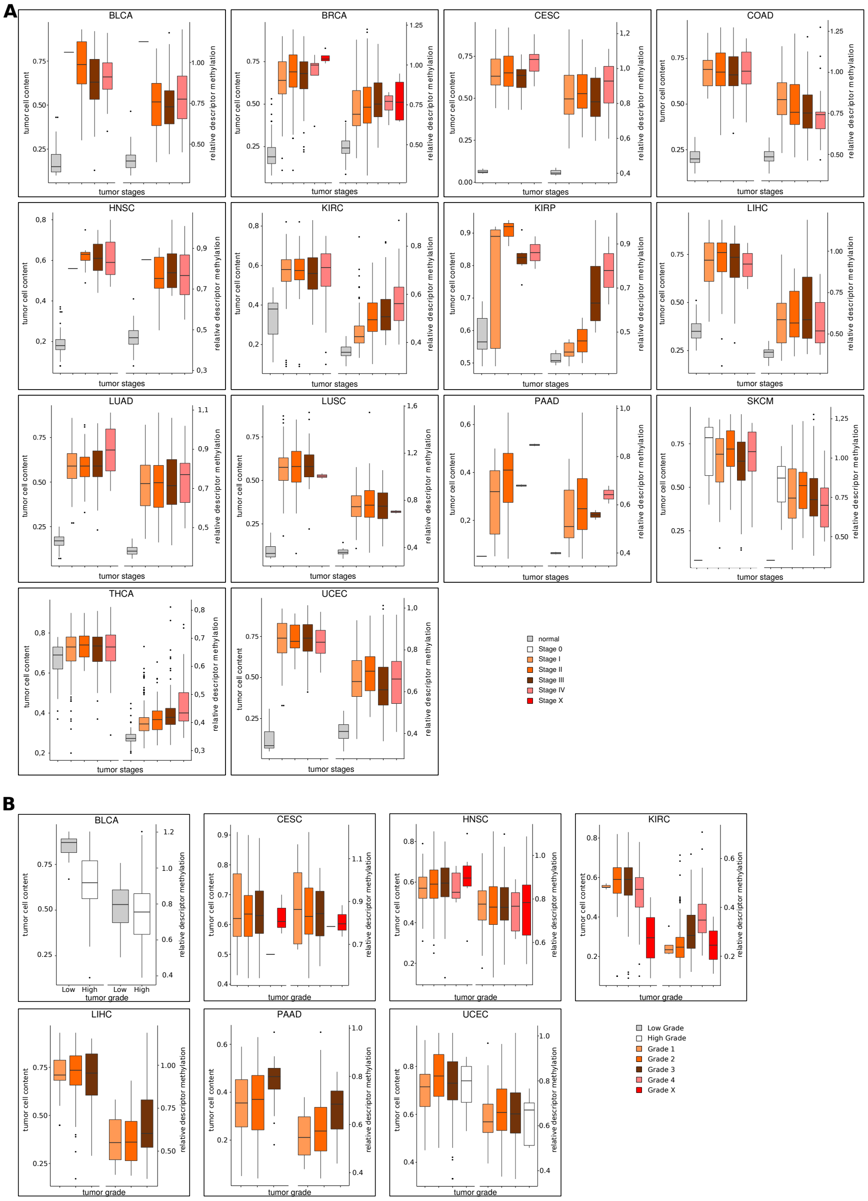

Supplemental Material, Figure S8: **A** Correlation of tumor cell content (left) and relative descriptor methylation (right) to pathologic tumor stages for 14 cancer types. **B** Correlation of tumor cell content (left) and relative descriptor methylation (right) to histologic tumor grades for 7 cancer types.

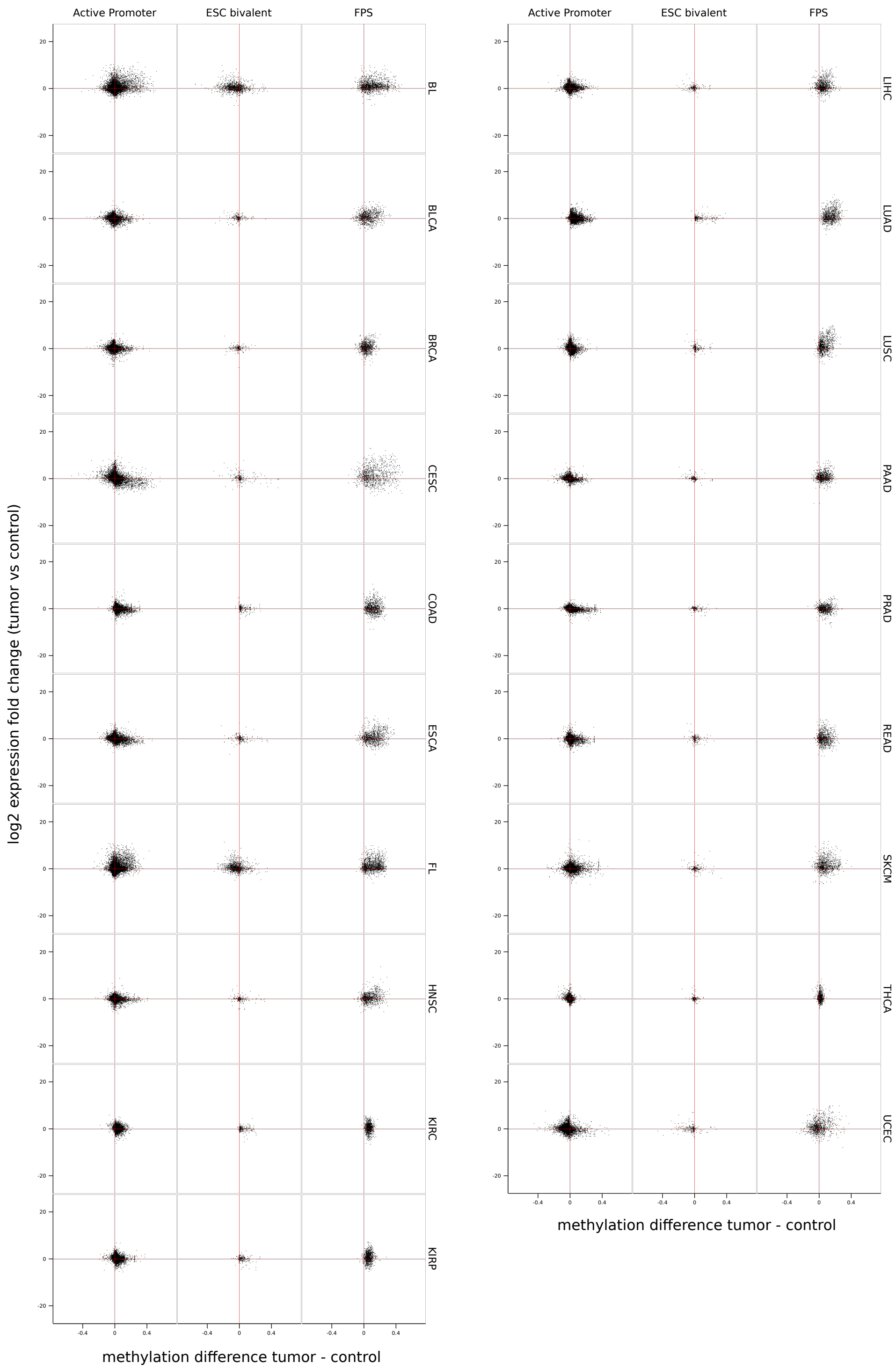

Supplemental Material, Figure S9: Correlation of methylation and expression change for all 19 cancer types. Correlation shown for active promoters (left) ESC based bivalent segments (center) and FBS (right).

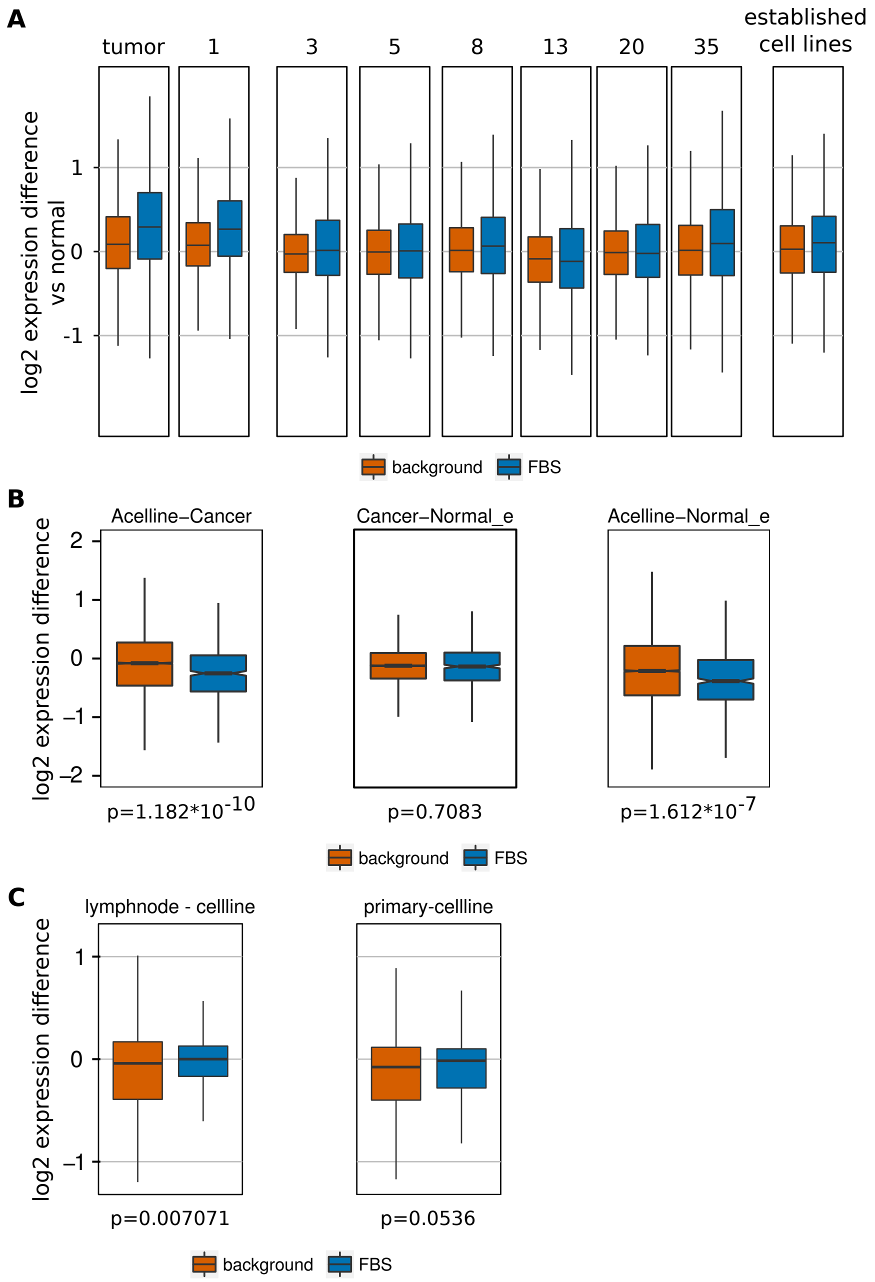

Supplemental Material, Figure S10: FBS and background gene expression change in cell lines. **A** FBS and background expression of glioblastoma cell lines grown in NBE. **B** Differences in expression between cervical cancer cell lines and fresh cervical cancer tissue (left), cell lines and normal micro dissected cervix epithelial tissue and cervical cancer and normal microdissected cervix epithelial tissue. **C** Expression difference between lung cancer cell lines from lymph node metastasis (left) and primary lung squamous cell tumor (right) and the cancers they originated from.

```
library(edgeR)
args <- commandArgs(trailingOnly = TRUE)
inname<-args[1]
data <- as.matrix(read.table(inname,row.names=1))
countData <- as.matrix(read.table(inname,header=T,row.names=1))
g<-as.vector(data[1,])
libSizes <- as.vector(colSums(countData))
d <- DGEList(counts=countData,group=g,lib.size=libSizes)
d <- calcNormFactors(d)
d <- estimateCommonDisp(d)
d <- estimateTagwiseDisp(d)
de.com <- exactTest(d)
results <- topTags(de.com,n = length(data[,1]))
write.table(as.matrix(results$table),file=paste(inname,"edgeR",
            "outputFile.txt",sep="."),sep="\t")
```

Supplemental Material, Figure S11: R-code used to compute differential expression with edgeR

Supplementary Tables

Supplemental Material, Table S1: Excel table of Roadmap tissues we used. Full table can be found at `https://docs.google.com/spreadsheet/d/1yikGx4MsO9Ei36b64yOy9Vb6oPC5IBGlFbYEt-N6gOM`

Supplemental Material, Table S2: Excel table of enriched GO-terms for bivalent elements, bivalent promoters, bivalent enhancers, FBS

Supplemental Material, Table S3: Excel table with accession numbers of data used.

Tumor type	tumor cell content (%)
BLCA	73
BRCA	77
CESC	36
COAD	73
HNSC	67
LUAD	47
LUSC	38
PAAD	27
SKCM	59
UCEC	75

Supplemental Material, Table S4: Estimated tumor cell content necessary for 5% false negative and 5% false positive rate.

Cancer type	Corr. to Tumor Stage	p	Corr to tumor Grade	p
COAD	-	0.0035		
KIRC	+	0.0070	+	4.24×10^{-5}
KIRP	+	0.0013		
LIHC			+	0.0215
THCA	+	0.0002		

Supplemental Material, Table S5: Correlation between relative descriptor methylation and pathologic tumor stage or histologic tumor grade, corrected for tumor cell content. Only significant correlations are shown. + means positive correlation, i.e. higher relative methylation at higher grades or stages.

Supplemental Material, Table S6: Excel table with significantly differentially expressed FBS genes. Subsets with genes assigned to different GO terms.

Supplemental Material, Table S7: Excel table with expression and methylation changes of FBS genes.

Gene	5'primer	3'primer	UPL Probe ID
DLX5	5-ctacaaccgcgtcccaag-3	5-gccattcaccattctcacct-3	#20
LEF1	5-cagatgtcaactccaaacaagg-3	5-ggagacaagggataaaaagtaggg-3	#17
PAX2	5-gagcacatcaaatcagaacagg-3	5-tgcagatagactcgacttgacttc-3	#23
SP8	5-ttaagagttattgttgcagttctgatg-3	5-aaaactaggccccgaaagtg-3	#21
ZIC2	5-gctccgagaacctcaagatcc-3	5-ccctcaaactcacactggaac-3	#7

Supplemental Material, Table S8: Primers and Universal ProbeLibrary IDS used for qPCR.
